# Supplementary material for: Phosphorylated tau181 in plasma as a potential biomarker for Alzheimer’s disease in adults with Down syndrome
Source: Nat Commun. 2021 Jul 14;12:4304. doi: 10.1038/s41467-021-24319-x (PMC8280160; doi:10.1038/s41467-021-24319-x)
Supplement: Supplementary file 1 — Supplementary Information [file 41467_2021_24319_MOESM1_ESM.pdf]

Supplementary Information

Supplementary Figure 1

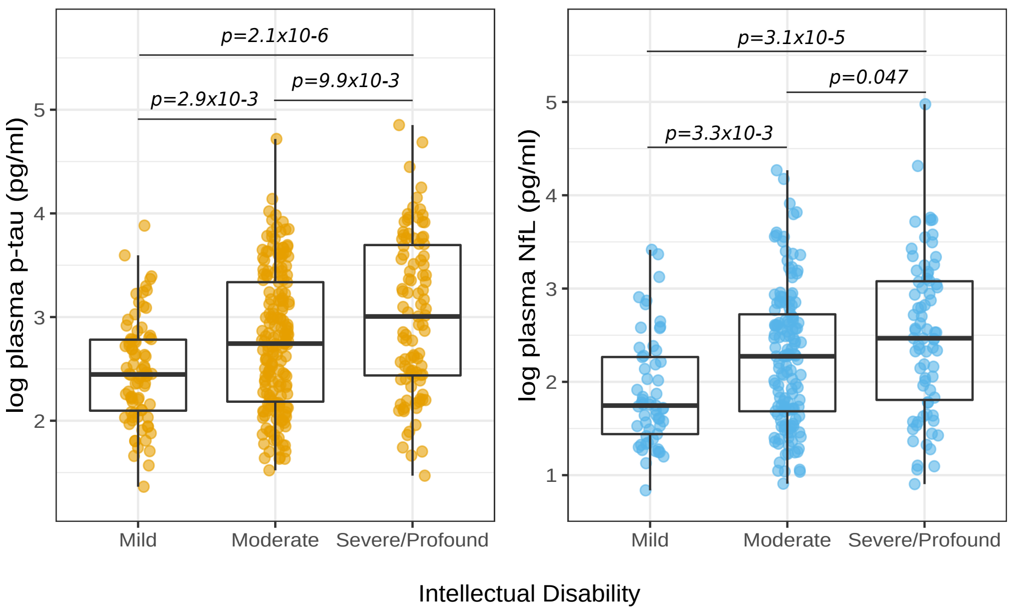

Plasma p-tau181 concentrations according to the degree of intellectual disability. a Concentrations of plasma p-tau plasma across mild (n=80), moderate (n=187) and severe/profound (n=99) intellectual disability. **b** Concentrations of plasma NfL across mild (n=57), moderate (n=147) and severe/profound (n=75) intellectual disability. The central black lines show the median values, regions above and below these lines show the upper and lower quartiles, respectively, and the whiskers extend from the IQR limits to the largest value no further than 1 • 5 × IQR. Two-sided Kruskal-Wallis with Dunn's test of multiple comparisons correcting by the Holm method was used to assess the differences between groups.

Supplementary Figure 2

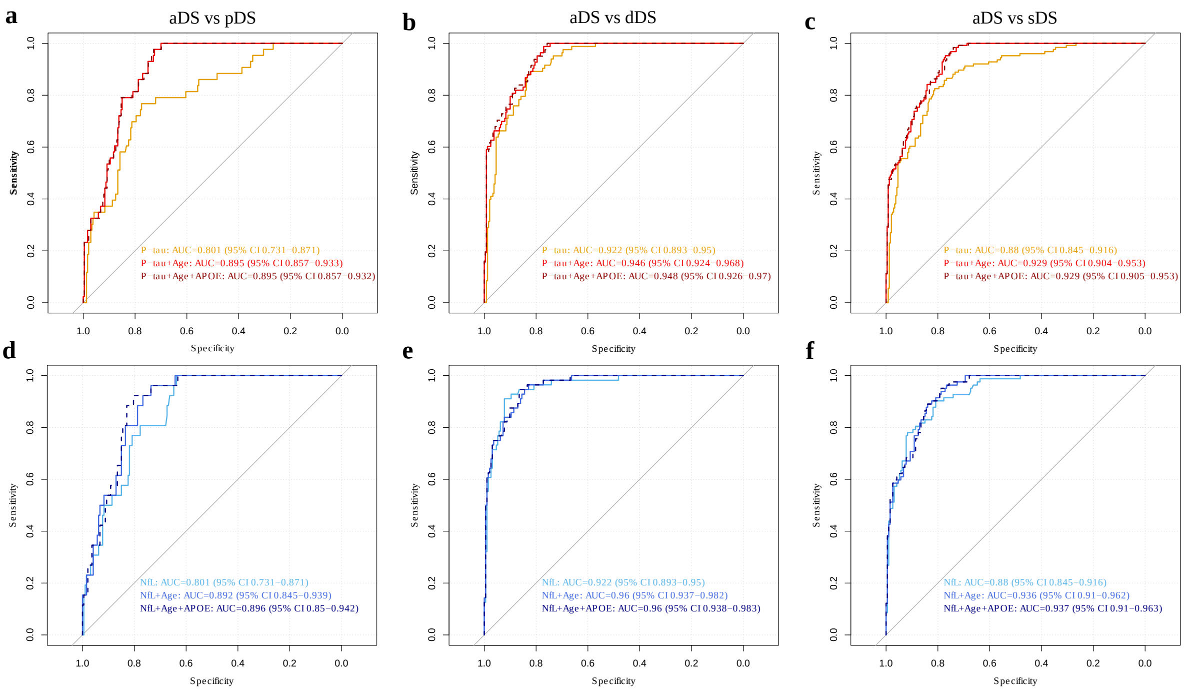

Diagnostic performance of p-tau and NfL, age, *APOE* genotype and their combination to detect AD in DS. **a** ROC curves for plasma p-tau to discriminate aDS and pDS. **b** aDS and dDS. **c** aDS and sDS. **d** ROC curves for plasma NfL to discriminate aDS and pDS. **e** aDS and dDS. **f** aDS and sDS. aDS: asymptomatic Down syndrome; pDS: prodromal Alzheimer's disease in Down syndrome; dDS: Alzheimer's disease dementia in Down syndrome; sDS: symptomatic Alzheimer's disease dementia in Down syndrome; AUC: area under the curve; CI: confidence interval.

Supplementary Figure 3

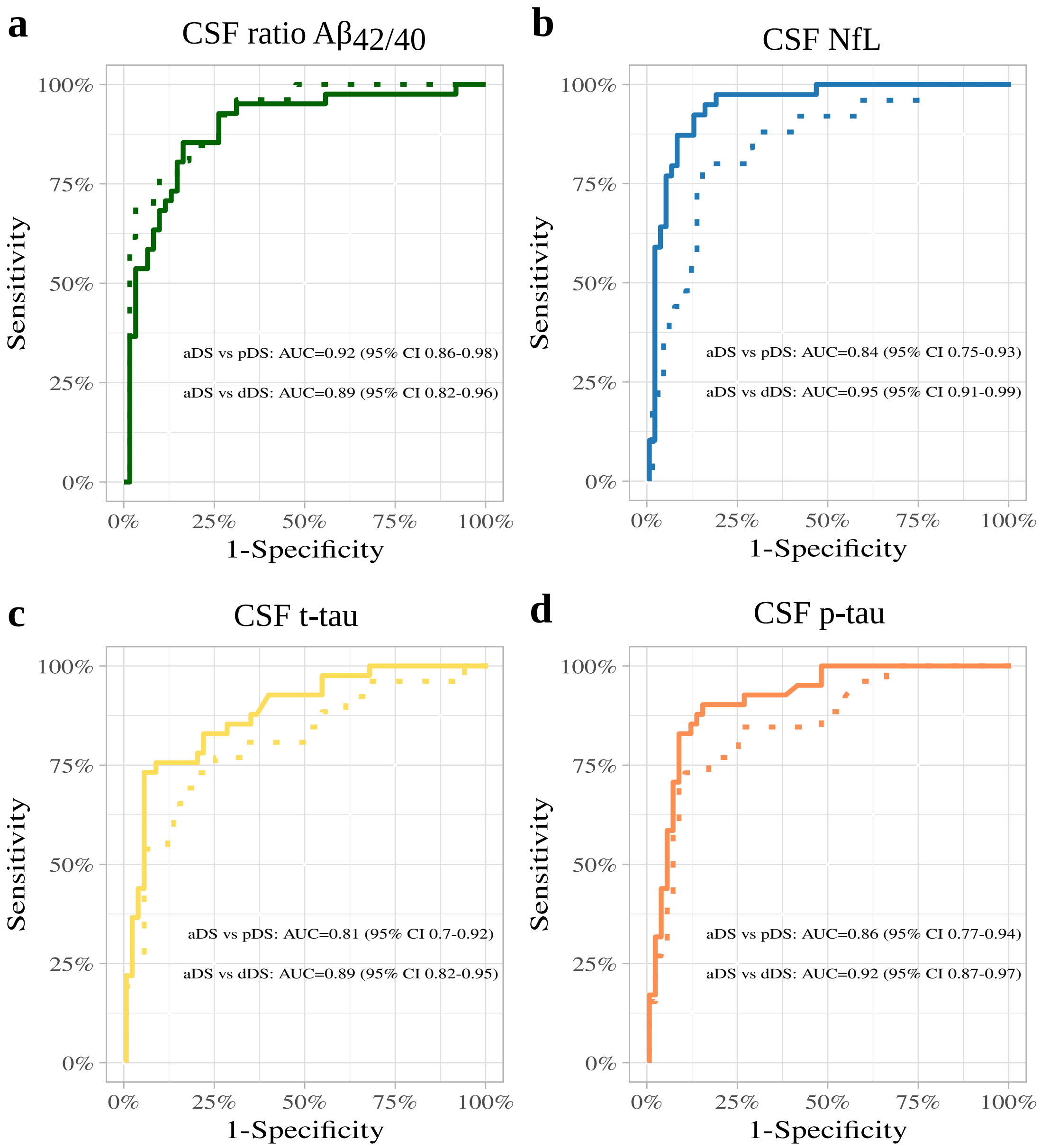

Diagnostic performance of CSF biomarkers to detect AD in DS. a ROC curves for CSF Aβ42/40 ratio to detect pDS and dDS from aDS. **b** ROC curves for CSF NfL to detect pDS and dDS from aDS. **c** ROC curves for CSF t-tau to detect pDS and dDS from aDS. **d** ROC curves for CSF p-tau181 to detect pDS and dDS from aDS. aDS: asymptomatic Down syndrome; pDS: prodromal Alzheimer's disease in Down syndrome; dDS: Alzheimer's disease dementia in Down syndrome; AUC: area under the curve; CI: confidence interval.

Supplementary Figure 4

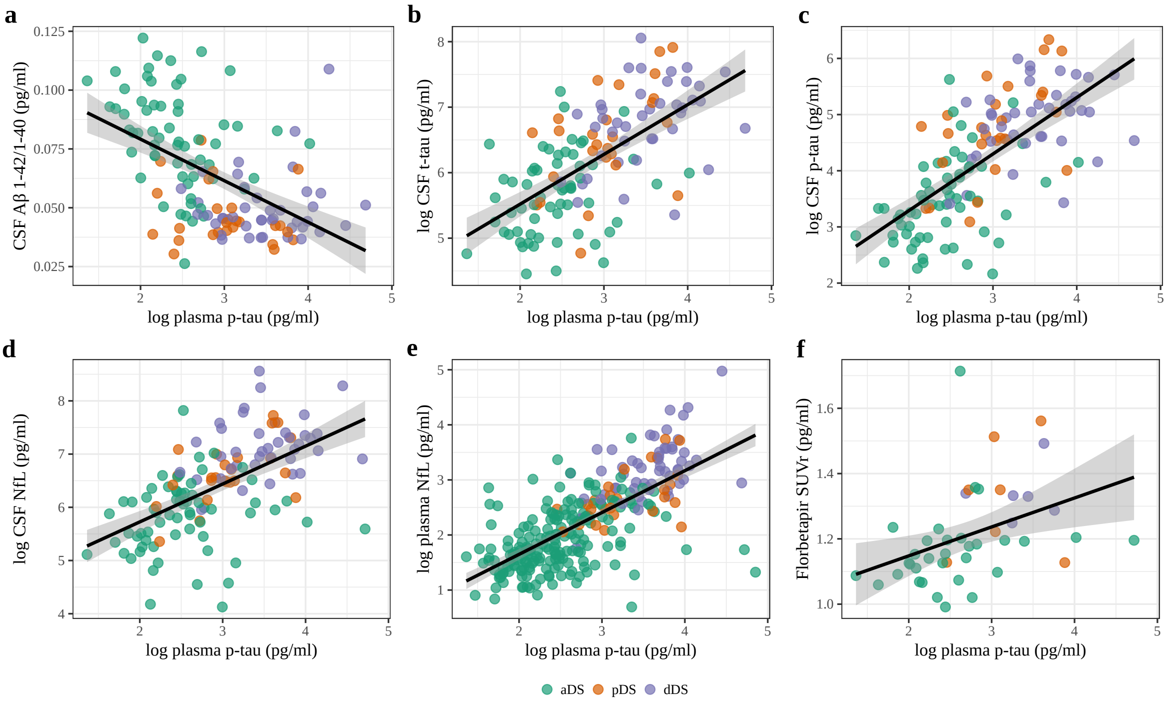

Age-adjusted partial correlation between plasma p-tau181 and different biomarkers. a CSF ratio Aβ42/40. **b** CSF t-tau. **c** CSF p-tau181. **d** CSF NfL. **e** Plasma NfL. **f** Florbetapir SUVr. The black lines indicate the fitted linear model and the shadowed ribbons show the 95% confidence level intervals.

Supplementary Table 1. Demographics, cognitive and biomarker data of participants with Down syndrome and controls with plasma p-tau181 and plasma NfL available (n=289).

	**Control**	**aDS**	**pDS**	**dDS**
N	14	193	26	56
Age (years) (median [IQR])	44.41 [37.96, 51.58]	37.96 [30.35, 45.24]	51.22 [47.90, 55.62]	53.62 [49.54, 56.72]
Gender = Male (%)	5 (35.7)	107 (55.4)	14 (53.8)	32 (57.1)
MMSE score (median [IQR])	30 [30, 30]	-	-	-
CAMCOG score (median [IQR])		78.00 [65.00, 86.00]	61.00 [43.25, 75.00]	41.50 [29.50, 55.00]
Degree of disability (%)				
Mild		52 (26.9)	4 (15.4)	1 (1.8)
Moderate		100 (51.8)	10 (38.5)	33 (58.9)
Severe/Profound		41 (21.2)	12 (46.2)	22 (39.3)
plasma p-tau181 (pg/ml) (median [IQR])	8.36 [6.82, 13.10]	11.37 [8.07, 16.05]	23.60 [19.18, 42.71]	38.70 [28.22, 46.75]
plasma NfL (pg/ml) (median [IQR])	3.38 [2.89, 4.16]	5.93 [4.43, 10.25]	13.61 [11.50, 18.26]	23.86 [17.33, 33.65]
CSF Aβ42/Aβ40 (median [IQR])	0.11 [0.10, 0.11]	0.08 [0.06, 0.10]	0.04 [0.04, 0.04]	0.05 [0.04, 0.05]
CSF t-tau (pg/ml) (median [IQR])	197.00 [168.75, 284.50]	275.00 [158.00, 464.00]	886.00 [593.50, 1472.25]	964.00 [671.00, 1541.75]
CSF p-tau (pg/ml) (median [IQR])	27.10 [21.88, 34.82]	31.90 [17.20, 58.70]	150.70 [97.95, 239.60]	155.00 [94.45, 224.60]
CSF NfL (pg/ml) (median [IQR])	304.00 [216.00, 356.45]	357.40 [212.78, 460.55]	751.33 [663.44, 1076.89]	1271.05 [924.52, 1631.50]
SUVR 18F-FBP (median [IQR])		1.15 [1.09, 1.20]	1.35 [1.17, 1.43]	1.33 [1.30, 1.34]
SUVR 18F-FDG (median [IQR])		1.34 [1.26, 1.40]	1.10 [1.06, 1.15]	0.78 [0.68, 0.90]

Abbreviations: N= sample, IQR= interquartile range, ID=intellectual disability, CSF= cerebrospinal fluid, Aβ =Amyloid-β, NfL= Neurofilament light protein, FDG= 18-Fluorodeoxyglucose, SUVR=Standardized Uptake Value Ratio. aDS= asymptomatic Down syndrome, pDS= prodromal Alzheimer's disease Down syndrome, dDS= Alzheimer's disease dementia Down syndrome, Controls= euploid healthy controls.
