# Supplementary material for: Phosphorylated tau181 in plasma as a potential biomarker for Alzheimer’s disease in adults with Down syndrome
Source: Nat Commun. 2021 Jul 14;12:4304. doi: 10.1038/s41467-021-24319-x (PMC8280160; doi:10.1038/s41467-021-24319-x)
Supplement: Supplementary file 2 — Reporting Summary [file 41467_2021_24319_MOESM2_ESM.pdf]

nature research

Corresponding author(s): ALBERTO LLEO/JUAN FORTEA

Last updated by author(s): May 20, 2021

Reporting Summary

Nature Research wishes to improve the reproducibility of the work that we publish. This form provides structure for consistency and transparency in reporting. For further information on Nature Research policies, see our Editorial Policies and the Editorial Policy Checklist.

Statistics

For all statistical analyses, confirm that the following items are present in the figure legend, table legend, main text, or Methods section.

n/a	Confirmed	
☐	☒	The exact sample size (n) for each experimental group/condition, given as a discrete number and unit of measurement
☐	☒	A statement on whether measurements were taken from distinct samples or whether the same sample was measured repeatedly
☐	☒	The statistical test(s) used AND whether they are one- or two-sided<br>*Only common tests should be described solely by name; describe more complex techniques in the Methods section.*
☐	☒	A description of all covariates tested
☐	☒	A description of any assumptions or corrections, such as tests of normality and adjustment for multiple comparisons
☐	☒	A full description of the statistical parameters including central tendency (e.g. means) or other basic estimates (e.g. regression coefficient) AND variation (e.g. standard deviation) or associated estimates of uncertainty (e.g. confidence intervals)
☐	☒	For null hypothesis testing, the test statistic (e.g. F, t, r) with confidence intervals, effect sizes, degrees of freedom and P value noted<br>*Give P values as exact values whenever suitable.*
☒	☐	For Bayesian analysis, information on the choice of priors and Markov chain Monte Carlo settings
☒	☐	For hierarchical and complex designs, identification of the appropriate level for tests and full reporting of outcomes
☐	☒	Estimates of effect sizes (e.g. Cohen's d, Pearson's r), indicating how they were calculated

Our web collection on statistics for biologists contains articles on many of the points above.

Software and code

Policy information about availability of computer code

Data collection	Freesurfer version 6, R software version 3.6.3.
Data analysis	All the statistical analyses were performed using R statistical software version 3.6.3.

For manuscripts utilizing custom algorithms or software that are central to the research but not yet described in published literature, software must be made available to editors and reviewers. We strongly encourage code deposition in a community repository (e.g. GitHub). See the Nature Research guidelines for submitting code & software for further information.

Data

Policy information about availability of data

All manuscripts must include a data availability statement. This statement should provide the following information, where applicable:

- Accession codes, unique identifiers, or web links for publicly available datasets
- A list of figures that have associated raw data
- A description of any restrictions on data availability

A statement about data availability has been included in the revised manuscript.

Field-specific reporting

Life sciences study design

All studies must disclose on these points even when the disclosure is negative.

Sample size	The study included the DABNI and SPIN cohorts. The DABNI cohort was based on a population-based health plan and all available plasma samples were analyzed in this study. Sample size was calculated based on previous biomarker studies that demonstrated enough statistical power. In particular, our previous study with smaller sample size (Fortea et al, Lancet Neurol 2018) showed adequate power.
Data exclusions	In 22 cases MRI studies were excluded due to movement artifacts or segmentation errors of the Freesurfer. In 4 cases FDG-PET images were excluded due to excessive time between MRI and PET.
Replication	The measurement of p-tau concentrations was perfomed using a robust immunoassay. All samples were measured at the first attempt.
Randomization	Randomization was not applicable as this study did not include any intervention.
Blinding	The technicians who measured concentrations of p-tau181 in plasma were blinded to the biomarker and clinical data. Investigators who removed imaging studies due to artifacts were blinded to clinical data. Blinding was not possible when obtaining clinical data.

Reporting for specific materials, systems and methods

We require information from authors about some types of materials, experimental systems and methods used in many studies. Here, indicate whether each material, system or method listed is relevant to your study. If you are not sure if a list item applies to your research, read the appropriate section before selecting a response.

Materials & experimental systems

n/a	Involved in the study	
☐	☒	Antibodies
☒	☐	Eukaryotic cell lines
☒	☐	Palaeontology and archaeology
☒	☐	Animals and other organisms
☐	☒	Human research participants
☒	☐	Clinical data
☒	☐	Dual use research of concern

Methods

n/a	Involved in the study	
☒	☐	ChIP-seq
☒	☐	Flow cytometry
☐	☒	MRI-based neuroimaging

Antibodies

Antibodies used	AT270 mouse monoclonal antibody (MN1050; Invitrogen, Waltham, MA, USA) as capture antibody.<br>Anti-tau mouse monoclonal antibody Tau12 (806502; BioLegend, San Diego, CA, USA) as the detector. It is not possible to report dilutions. Full details can be found in Karikari et al. Lancet Neurol 2020; 19: 422–33
Validation	Full validation of the assay was described in Karikari et al. Lancet Neurol 2020; 19: 422–33

Human research participants

Policy information about studies involving human research participants

Population characteristics	Adults with DS (age range 18-71) and euploid controls (age range 21-55). Both genders were included.
Recruitment	Adults with DS were recruited from a population-based health plan designed to screen for AD dementia, which includes yearly neurological and neuropsychological assessments. We acknowledge that this study may be biased towards subjects with DS and mild/moderate disability as subjects with profound intelectual disability have difficulties in access to research studies. Those individuals interested in research studies are included in the<br>longitudinal cohort. We recruited the euploid controls from the Sant Pau Initiative on Neurodegeneration cohort.
Ethics oversight	The study was approved by the Sant Pau Ethics Committee, following the standards for medical research in humans recommended by the Declaration of Helsinki.

Note that full information on the approval of the study protocol must also be provided in the manuscript.

Magnetic resonance imaging

Experimental design

Design type	The study included structural MRI measurements.

Design specifications	Each participant underwent a 45 min MRI session.
Behavioral performance measures	There were no behavioral measures in this study.

Acquisition

Imaging type(s)	Structural
Field strength	3
Sequence & imaging parameters	MPRAGE protocol; RAS orientation; 160x256x256 slices, voxel size 1x0.94x0.94mm; TR=8.1ms; TE=3.7ms; Flip Angle= 8
Area of acquisition	Whole brain scan

Diffusion MRI ☐ Used ☒ Not used

Preprocessing

Preprocessing software	Freesurfer 6.0 was used to process the T1w. The skull is stripped using a deformable template model. Voxels are classified as white matter based on intensity and neighbour constraints and a white matter/gray matter surface is then generated for each hemisphere. This surface is then refined using intensity gradients and a watershed is appplied to extract the gray matter/CSF boundary. The cortical thickness is computed as the distance between both surfaces. Smoothing kernel of 15mm (in surface) was applied to the cortical thickness maps.
Normalization	The T1 is first registered to the MNI305 space using an affine registration. Then, a high dimensional nonlinear volumetric alignment to the MNI305 atlas is performed. Finally, the MNI estimated surfaces are projected to a standard spherical space.
Normalization template	Spherical space (fsaverage) as implemented in Freesurfer was used as a standard space.
Noise and artifact removal	Only movement artifacts were taking into account to exclude subjects.
Volume censoring	Only surface-based estimations were assessed as this work's aim is to track cortical changes

Statistical modeling & inference

Model type and settings	Mass univariate analysis was performed at cortical level. Only fixed effects were used.
Effect(s) tested	To test the associations, only linear models were used.

Specify type of analysis: ☒ Whole brain ☐ ROI-based ☐ Both

Statistic type for inference (See Eklund et al. 2016)	Vertex-wise analysis was performed and $p<0.05$ was set at vertex level
Correction	Monte-Carlo simulation with 10,000 repeats as implemented in Freesurfer (family-wise error [FWE] correction at $P<0.05$) was tested.

Models & analysis

n/a	Involved in the study
☒	☐ Functional and/or effective connectivity
☒	☐ Graph analysis
☐	☒ Multivariate modeling or predictive analysis

Multivariate modeling and predictive analysis	General linear model was used to predict cortical thickness (mm) or FDG-PET uptake (SUVr) with plasma p-tau levels (pg/ml).
